# Use of Point-of-care Ultrasound to Diagnose Rectus Abdominis Strain in the Acute Setting: A Case Report

**DOI:** 10.5811/cpcem.1670

**Published:** 2024-03-26

**Authors:** Alejandro J. Sanoja, Michael Shalaby

**Affiliations:** Mount Sinai Medical Center, Department of Emergency Medicine, Miami Beach, Florida

**Keywords:** *rectus abdominis*, *ultrasound*, *muscle tear*, *emergency department*, *case report*

## Abstract

**Introduction:**

Rectus abdominis muscle strains are common and can be debilitating in both professional and amateur athletes who engage in strenuous activity.

**Case Report:**

We report a rare case of rectus abdominis muscle tear in an amateur bodybuilder diagnosed by point-of-care ultrasound (POCUS) in the emergency department (ED). The patient had presented to the ED three separate times after strenuous exercise, received costly diagnostic workups, and ultimately was diagnosed on the third visit with grade 2 bilateral rectus abdominis tear. The patient was given appropriate education and sports medicine follow-up. He underwent rehabilitation focused on trunk and core stability. At eight-week follow-up, the patient had made a full recovery.

**Conclusion:**

To our knowledge, a case of bilateral rectus abdominis tear diagnosed by ultrasound in the emergency setting has not been previously published. Our case report highlights the utility of POCUS in diagnosing musculoskeletal pathology and preventing costly bounce-back visits.

Population Health Research Capsule What do we know about this clinical entity?

*Rectus abdominis muscle strains are easily misdiagnosed in the emergency setting. point of care ultrasound (POCUS) has been shown to aide in diagnosis in the sports medicine literature.*

What makes this presentation of disease reportable?

*There has never been a reported case of rectus abdominis strain that was diagnosed by POCUS in the emergency medicine literature.*

What is the major learning point?

*This case report emphasizes how POCUS is an important, sensitive, and cost-effective aide in evaluating abdominal wall pathology.*

How might this improve emergency medicine practice?

*This may encourage emergency medicine physicians to routinely use POCUS when suspecting musculoskeletal pathology.*

## INTRODUCTION

Rectus abdominis muscle strains are common and debilitating in both professional and amateur athletes but can be easily misdiagnosed in an emergency setting or by physicians who do not treat athletes regularly.^1^^–^^10^ Point-of-care ultrasound (POCUS) is a helpful tool to aid in expedient diagnosis and grade the degree of severity of rectus abdominis strain.^7^ Here we describe a case of rectus abdominis muscle strain that highlights the utility of POCUS, the importance of discriminating between intra-abdominal and abdominal wall pathology, and the prevention of poor functional outcomes.

## CASE REPORT

The patient was a 40-year-old male recreational bodybuilder with no significant prior medical history who presented to the emergency department (ED) with lower abdominal pain. He trained five days a week with a focus on weightlifting and included a variety of endurance exercises in his regimen. Of note, he had recently increased his endurance training and core isolation exercises.

At the patient’s first ED visit, he complained of debilitating abdominal pain that was located below the umbilicus, constant and progressively worsening. His pain was aggravated by running, abdominal isolation exercises, and deadlifting. The patient had not tried any medications to alleviate the pain. He denied any constitutional symptoms, dysuria, hematuria, vomiting, or diarrhea. Physical exam was significant for severe suprapubic tenderness to palpation. Lab work was within normal limits, and computed tomography (CT) of the abdomen and pelvis with oral and intravenous contrast revealed no abnormalities. The patient was given ondansetron, normal saline, viscous lidocaine, and famotidine and was discharged home with primary care follow-up.

The patient returned three months later with persistent lower abdominal pain. He reported that his aggravating and alleviating factors had not changed, but that his abdominal pain had progressed. He had not followed up with a primary care physician or any other specialist since his first visit. He once again had labs drawn and CT abdomen and pelvis imaging, which were again within normal limits. The patient was again given pain medicines and was discharged home with a diagnosis of “unspecified lower abdominal pain,” and given follow-up with gastroenterology and urology.

**Image 1.**
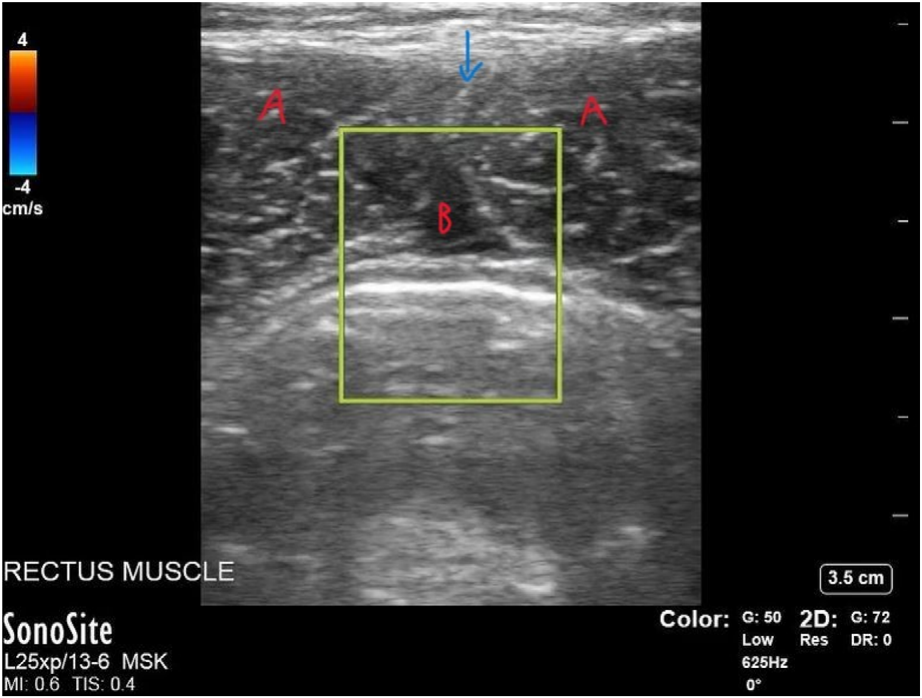
Point-of-care ultrasound of the rectus abdominus in short-axis view demonstrating a small, 1 × 2-centimeter collection of fluid. In the near field is skin and subcutaneous tissue. The hyperechoic midline stripe between the two triangular rectus abdominis muscle bellies (“A”) on either side is the linea alba (blue arrow). The hypoechoic area (labeled “B”) between the rectus abdominis muscles and below the linea alba is likely edema. The yellow rectangle is a color flow application, which indicates arterial (red) or venous (blue) blood flow. In this case, there is no flow, which is expected in the case of edema.

**Image 2.**
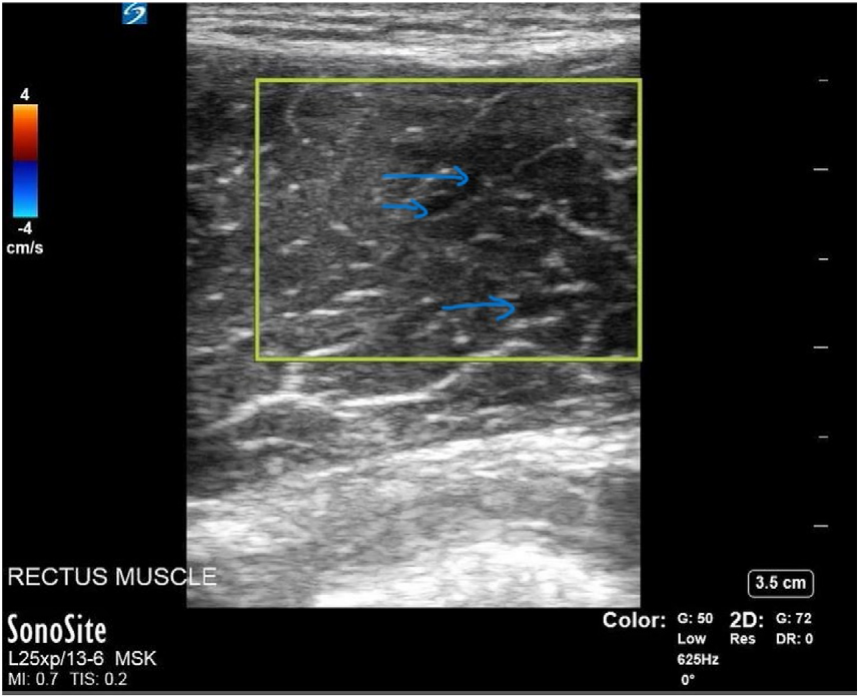
Point-of-care ultrasound of the right rectus abdominus muscle in the short-axis view demonstrating muscle fiber elongation with intramuscular edema indicated by the blue arrows. Again, the yellow rectangle demonstrates no evidence of small vascular structures.

The patient presented again after another three months with continued abdominal pain, which he noted still occurred after the same exacerbating exercises from prior. Physical examination was significant for pain elicited with flexion of the abdominal muscles and palpation. Additionally, the patient was evaluated by soft tissue ultrasound over the most painful area of the abdomen. Point-of-care ultrasound (POCUS) of the abdominal rectus muscle demonstrated disrupted abdominal muscle fibers bilaterally with anechoic fluid between abdominis muscle layers (Image 1) and streaks of edema intramuscularly (Image 2). The emergency physician diagnosed the patient with rectus abdominis strain and instructed him to rest and follow up with a sports medicine physician.

The patient was contacted two months after discharge from the ED. He had stopped performing the culprit exercises until his abdomen no longer hurt. He modified aggravating isolation abdominal exercises and progressively over the course of two months returned to his previous normal activity without pain.

## DISCUSSION

Abdominal rectus injuries are overall a rare entity in the general population; however, they occur in physically active individuals who engage in frequent strenuous exercise.^1^ The literature describes these injuries in a diversity of professional and recreational athletes, including recreational weightlifters.^1^^–^^9^ Clinically, there are notable historical and examination findings that are helpful to distinguish abdominal wall from visceral pathology. Abdominal wall injuries tend to be more localized, elicited by movement, and are tender to palpation regardless of muscle contractility. The Carnett sign is elicited when the point of maximal abdominal tenderness persists when the patient’s abdomen is palpated while tensed. If the Carnett sign is positive, then the pain is likely to arise from the abdominal wall.^1^ Visceral pain can be more diffuse, referred, constant, and is elicited when the muscles are relaxed. Additionally, there is a notable absence of nausea, vomiting, diarrhea, constitutional symptoms, or other symptoms common in intra-abdominal pathologies.^1^ As in this case, the presentation can be ambiguous, and POCUS is a powerful tool that can be used to aid in diagnosis.

In this case, both improved physical exam and ultrasound were invaluable in reliably diagnosing the patient with rectus abdominis muscle tear and preventing further delay in treatment. A few case reports highlight ultrasound as a tool to both diagnose and treat abdominal muscle tears.^1^^–^^7^^,^^9^ Ruff et al and Maquirriain et al recommend using ultrasound to estimate disease severity. The goal of ultrasonography in the setting of suspected muscular pathology is to identify a tear, as this prognosticates time to recovery and to sport.^7^ Tears are characterized by disturbed muscle fibers, intramuscular edema, and fluid collections. As with our patient, rectus abdominis strain can be easily overlooked when a patient is in acute distress due to pain. The patient presented three times to the ED before a correct diagnosis was made. This can lead to unnecessary testing and inappropriate treatment.

Revision of CT imaging by an attending emergency physician after ultrasound diagnosis showed no significant acute abnormality. Revision by a radiologist was not performed, but the second CT report included a revision of the first scan for comparison. Both CT studies, read by two different radiologists, showed no acute abdominal wall pathology. Ultrasound and magnetic resonance imaging are preferred tests to evaluate for muscular pathology due to increased sensitivity and specificity.^1^^,^^2^^,^^10^ Computed tomography has limited utility for muscular lesional evaluation but will assess for fracture and intrabdominal pathology. As discussed above, ultrasound is both specific and sensitive in detecting muscle tears.^10^ Magnetic resonance imaging can provide additional information and is typically ordered for the professional athlete. However, numerous studies have shown POCUS to be both sensitive and specific in detecting muscular pathology and has the added benefit of being cost-effective and rapidly available in most EDs.^4^^,^^7^^,^^10^

Treatment for most rectus abdominal tears is conservative. The cornerstone of treatment in the acute period is rest, ice, analgesia, and avoidance of the offending action.^1^^,^^5^ Early rehabilitation is considered key to reducing time to play.^5^ Rehabilitation focuses on functional restoration exercises.^1^^,^^5^^–^^8^ Several case reports support the use of platelet rich therapies and corticosteroid injection in professional athletes in addition to rehabilitation.^3^^,^^4^ The patient discussed here improved with conservative management and a progressive return to weightlifting.

## CONCLUSION

Identification of abdominal wall injury is easily overlooked by acute care physicians, but it is crucial to prevent delayed treatment, poor functional outcomes, and costly medical workup. This case demonstrates how ultrasonography can be used to diagnose abdominal muscle tears in both the ED and outpatient clinic. Ultrasound is reliable, quick, and cost-effective. To our knowledge, there are fewer than 12 case reports on this topic, with none occurring in the ED that resulted in real-time diagnosis. We support the routine use of POCUS to evaluate musculoskeletal pathology in the ED and in the outpatient setting.
